# Analysis of differential gene expression in human melanocytic tumour lesions by custom made oligonucleotide arrays

**DOI:** 10.1038/sj.bjc.6602612

**Published:** 2005-05-17

**Authors:** N J W de Wit, J Rijntjes, J H S Diepstra, T H van Kuppevelt, U H Weidle, D J Ruiter, G N P van Muijen

**Affiliations:** 1 Department of Pathology, Radboud University Nijmegen Medical Centre, Nijmegen, The Netherlands; 2 Department of Biochemistry, Radboud University Nijmegen, Nijmegen, The Netherlands; 3 Roche Diagnostics GmbH, Pharma Research, Penzberg, Germany

**Keywords:** melanoma, differential expression, tumour progression, oligonucleotide array, real-time PCR

## Abstract

Melanoma is one of the most aggressive types of cancer and resection of the tumour prior to dissemination of tumour cells is still the most effective treatment. Therefore, early diagnosis of melanocytic lesions is important and identification of novel (molecular) markers would be helpful to improve diagnosis. Moreover, better understanding of molecular targets involved in melanocytic tumorigenesis could possibly lead to development of novel interventions. In this study, we used a custom made oligonucleotide array containing 298 genes that were previously found to be differentially expressed in human melanoma cell lines 1F6 (rarely metastasising) and Mel57 (frequently metastasising). We determined differential gene expression in human common nevocellular nevus and melanoma metastasis lesions. By performing nine dye-swap array experiments, using individual as well as pooled melanocytic lesions, a constant differential expression could be detected for 25 genes in eight out of nine or nine out of nine array analyses. For at least nine of these genes, namely THBD, FABP7, H2AFJ, RRAGD, MYADM, HR, CKS2, NCK2 and GDF15, the differential expression found by array analyses could be verified by semiquantitative and/or real-time quantitative RT–PCR. The genes that we identified to be differentially expressed during melanoma progression could be potent targets for diagnostic, prognostic and/or therapeutic interventions.

Melanoma is a very aggressive type of tumour, as it metastasises early in tumour progression. Due to its relative insensitivity to systemic therapies, such as chemotherapy and radiation, the most effective cure for melanoma patients nowadays remains surgical excision of the tumour before onset of the metastatic growth phase. This means that early diagnosis of melanocytic tumour lesions is essential.

The molecular mechanisms underlying malignant transformation of melanocytes and melanoma tumour progression are not very complete yet. Extensive analysis of molecular changes that occur during tumour development may not only provide better insight in melanocytic tumorigenesis, but additionally yield valuable tools for clinical applications. Novel diagnostic and/or prognostic markers and targets for (immuno-)therapy could be identified by determining the differential gene expression in different stages of melanoma progression.

For most microarray studies related to melanocytic tumorigenesis, cell lines or fresh tumour cells that were cultured for some passages were used to examine differential gene expression (Baldi *et al*, 2003a; Dooley *et al*, 2003; Rumpler *et al*, 2003; Hoek *et al*, 2004). However, usage of these cells is not ideal, as culturing conditions can influence the genetic expression. In a previous study, we also determined differential gene expression in two human melanoma cell lines, 1F6 and Mel57, showing distinct metastatic behaviour after subcutaneous inoculation into nude mice, by using high-density oligonucleotide array analyses (Affymetrix) (Westphal *et al*, 1997; de Wit *et al*, 2002). We found an up- or downregulation of 298 genes/ESTs among the more than 40 000 genes that were analysed. In this study, we now used fresh human melanocytic tumour lesions of different progression stages to determine whether the previously identified genes remained differentially expressed in the *in vivo* situation. This would probably be more informative regarding involvement of the genes in melanoma progression. Custom oligonucleotide arrays were designed representing the 298 genes/ESTs and hybridisation was performed using target probes derived from common nevocellular nevus (NN) and melanoma metastasis (MM) samples. After array analyses, differential gene expression was verified by semiquantitative and real-time quantitative reverse transcriptase (RT)–PCR. The reliability of our custom array analyses is discussed, next to the putative involvement of the differentially expressed genes in melanoma tumour progression and their potential significance as new diagnostic/prognostic markers and/or targets for (immuno-) therapy.

## MATERIALS AND METHODS

### Human tissue samples and cell lines

Human melanocytic tumour samples were obtained by resection of the lesions at University Medical Centre (UMC) St Radboud Nijmegen (The Netherlands). This was all performed according to local ethical guidelines and approved by the local regulatory committee. After resection, all tissue samples were immediately frozen in liquid nitrogen and stored at −80°C until use.

Human melanoma cell lines, 1F6 and Mel57, were grown in Dulbecco's modified Eagle's medium (DMEM) as described previously (de Vries *et al*, 1996; Westphal *et al*, 1997).

### Design and printing of custom oligonucleotide arrays

**Figure 1:**
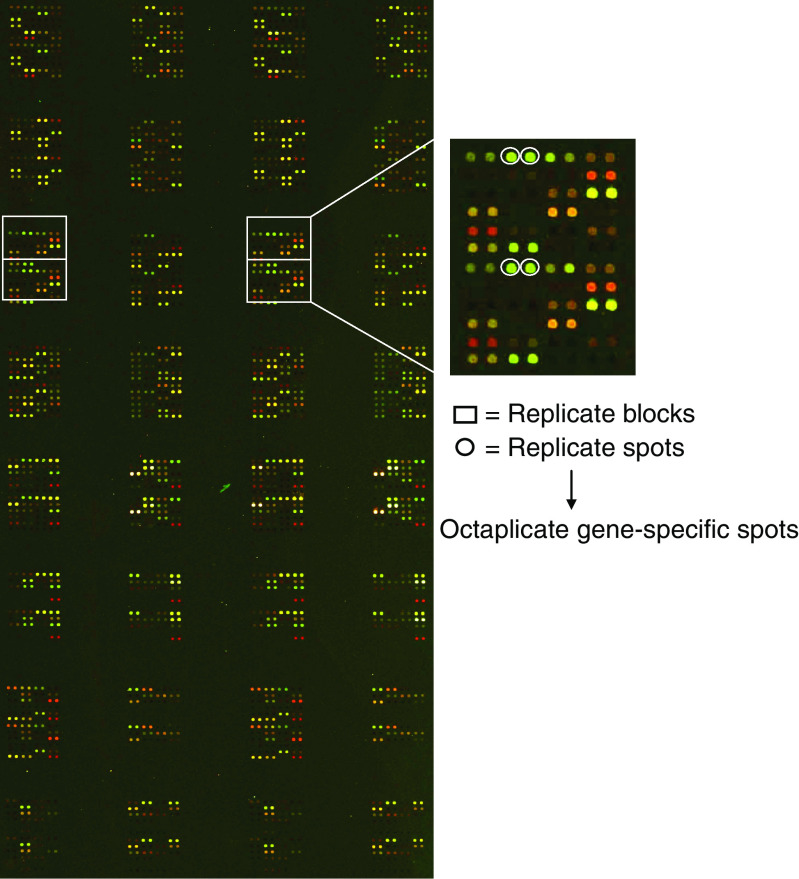
Custom made oligonucleotide array design. 70-mer oligo's, corresponding to 298 genes that were previously found to be differentially expressed in cell lines 1F6 and Mel57 and four additional housekeeping genes, were spotted in octaplicate on UltraGAPS slides. The housekeeping genes were randomly localised throughout the array. Additionally, various other controls for labelling and hybridisation efficiency were present on the array, such as oligo's of non-human species, Cot-1 repetitive sequences, polyA sequences and spots containing only spotting buffer.

In a previous study, 298 genes/ESTs were identified showing differential gene expression in two human melanoma cell lines, 1F6 and Mel57 (de Wit *et al*, 2002). For preparation of custom arrays these genes/ESTs were selected, together with four housekeeping genes, namely glyceraldehyde-3-phosphate dehydrogenase (GAPDH), phosphoglycerate kinase 1 (PGK1), porphobilinogen deaminase (PBGD) and *β*-actin. Based on these genes/ESTs, 70-mer amino-linked oligonucleotides were designed (Operon Technologies Inc., Qiagen, Alameda, CA, USA), complementary to the 3′-side of the corresponding genes/ESTs. These oligonucleotides were dissolved in spotting buffer (3 × SSC, 1.5 M betaine) and spotted in octaplicate onto UltraGAPS slides (Corning, New York, USA) using the Prosys 5510TL arrayer (Genomic Solutions, Huntingdon, Cambridgeshire, UK) (Figure 1). Additionally, various controls, which provided information about the target probe labelling efficiency, blocking, and nonspecific binding of the arrays, were spotted onto the array. These controls included oligonucleotides of various non-human species, Cot-1 repetitive sequences, polyA sequences (SpotReport, Stratagene, La Jolla, CA, USA), tRNA and spots containing only spotting buffer. Supplementary array design data are available at the NCBI Gene Expression Omnibus, GEO (www.ncbi.nlm.nih.gov/geo/).

### RNA isolation

For total RNA extraction from tissue samples, at least 10 frozen slices of 20 *μ*m thickness were collected in 1 ml TRIzol Reagent (Invitrogen, Carlsbad, CA, USA). After the TRIzol method, total RNA was subjected to an additional RNeasy (Qiagen, Hilden, Germany) cleaning step. Concerning the cell lines, total RNA was isolated from 10^7^ cultured cells using the RNeasy mini kit (Qiagen). All methods were performed conform the manufacturer's protocol.

### Target labelling, hybridisation and array analysis

Both direct and indirect labelling methods were used for labelling of target probes. Using the CyScribe first-strand cDNA labelling kit (Amersham Biosciences, Freiburg, Germany), cDNA was directly labelled by incorporation of Cy3- and Cy5-dUTPs during a RT reaction. We first isolated mRNA from 50 *μ*g of total RNA using the Oligotex mRNA mini kit (Qiagen). The subsequent labelling reaction was performed according to the manufacturer's protocol. For indirect labelling, 2 *μ*g of total RNA (ratio 28S/18S RNAs>1) were subjected to linear RNA amplification using the Amino Allyl MessageAmp aRNA Kit (Ambion, Cambridgeshire, UK). During this procedure, 5-(3-aminoally)-UTP was incorporated into the amplified antisense RNA (aRNA). *N*-hydroxysuccinimidyl (NHS) ester-derivitised reactive Cy3 and Cy5 dyes (Fluorolink Cy3/Cy5 Monofunctional Dye 5-Pack, Amersham Biosciences) were then chemically coupled to 3 *μ*g of amino allyl aRNA. RNA amplification as well as chemical labelling was performed according to the instruction manual of the Amino Allyl MessageAmp aRNA Kit. Cy3 and Cy5 dye incorporation was measured by spectrophotomeric analyses at 550 and 650 nm, respectively.

Prior to hybridisation, Cy3 and Cy5 labelled cDNA/aRNA samples were mixed (1 : 1), together with 7 *μ*g Cot-1 DNA (Roche Diagnostics) and 3 *μ*g polyA (Amersham Biosciences). After precipitation, the sample was dissolved in 130 *μ*l of a hybridisation solution containing 50% formamide, 10% dextran sulphate, 2 × SSC, 4% SDS, and 10 *μ*g/*μ*l yeast tRNA (Invitrogen). Hybridisation to our custom arrays and posthybridisation washing procedures were performed using a GeneTAC Hybridization Station (Genomic Solutions), according to the manufacturer's protocols. In short, a 16 h hybridisation with active circulation of the probe was followed by five posthybridisation wash cycles in 50% formamide/2 × SSC at 45°C and five wash cycles in PBS at 20°C. The slides were briefly washed in water and dried by centrifugation. In dye-swap experiments, two replicate arrays were hybridised with similar cDNA/aRNA samples, but with swapped dyes.

Arrays were scanned and imaged on an Affymetrix 428 scanner (Affymetrix, Santa Clara, CA, USA) using the Affymetrix 428 scanner software package (version 1.0). Generally, the Cy5 dye showed a higher fluorescence intensity than the Cy3 dye. Therefore, scanning intensities used to image the individual dyes were adjusted to obtain a Cy5/Cy3 ratio=1 for the housekeeping genes GAPDH and *β*-actin. To determine differential gene expression, the acquired array images were first analysed visually (red or green spots), but additionally GenePix Pro 4.0 software (Axon Instruments, Union City, CA, USA) was used. In the latter, differential gene expression was determined by comparing the median of the pixel intensities minus the median local background (F-B) of Cy5 with that of Cy3. Spots displaying F-B values smaller than 100 for both Cy dyes were excluded from further analysis. Alternatively, differential gene expression was determined by transforming median of ratios values (GenePix Pro 4.0) by taking the log_2_. All octaplicate gene-specific spots were included in the analyses. Supplementary microarray data can be found in the NCBI Gene Expression Omnibus, GEO (www.ncbi.nlm.nih.gov/geo/).

### Semiquantitative RT–PCR

Aliquots of 1 and 0.5 *μ*g of total RNA from cell lines and human melanocytic tissue samples, respectively, were reverse-transcribed using Moloney murine leukaemia virus reverse transcriptase (M-MLV RT) (Promega, Madison, WI, USA). Apart from 200 U of M-MLV RT, the reaction mixture consisted of 250 pmol of random hexadeoxynucleotide primers (Roche Diagnostics GmbH, Penzberg, Germany), 4 *μ*l of RT buffer (250 mM Tris-HCl pH 8.3, 375 mM KCl, 15 mM MgCl_2_, 50 mM DTT) and 4 *μ*l of 1 mM dNTPs (Roche Diagnostics GmbH), completed with water to a final volume of 20 *μ*l. This mixture was incubated 10 min at 25°C, 59 min at 42°C and 5 min at 95°C. cDNA samples of human melanocytic lesions were diluted 1 : 1 in water.

Generally, PCR amplification was carried out in a total volume of 25 *μ*l containing 1 *μ*l reverse-transcribed cDNA, 2.5 *μ*l of PCR buffer IV (20 mM (NH_4_)_2_SO_4_, 75 mM Tris/HCl pH 9.0 and 0.1% Tween), 5 pmol of each primer, 0.15 U of Thermoperfectplus DNA polymerase (Integro, Zaandam, The Netherlands) and the appropriate MgCl_2_ concentration. After 5 min denaturation at 94°C, 25/30 and 30/35 cycles of amplification were carried out for cell lines and human melanocytic lesions, respectively: 45 s at 94°C, 45 s at 59°C, 1 min at 72°C, followed by a 5-min elongation step at 72°C. Housekeeping genes GAPDH and *β*-actin that were used for normalisation were amplified in 25/30 cycles independent of cDNA origin. The primer sequences and MgCl_2_ concentrations used for each single PCR reaction are shown in Table 1.

### Real-time quantitative RT–PCR

To determine differential gene expression of fatty acid binding protein 7 (FABP7, UniGeneID Hs.26770) and growth differentiation factor 15 (GDF-15, UniGene ID Hs.296638) by real-time quantitative RT–PCR (qPCR), we used Assays-on-Demand Gene Expression Assays (Applied Biosystems, Cheshire, UK); assay ID Hs00361426_m1 and Hs00171132_m1, respectively. These assays consisted of a mix of unlabelled PCR primers and TaqMan MGB probe (FAM dye-labelled). Additionally, for normalisation, Assays-on-Demand Gene Expression Assay specific for GAPDH (assay ID Hs99999905_m1) was selected. The efficiency of each assay was tested in a cDNA dilution series. qPCR reactions were performed in a total volume of 25 *μ*l, containing 2 × TaqMan Universal PCR Master Mix (Applied Biosystems), 20 × Assays-on-Demand Gene Expression Assay Mix and 12.5 ng cDNA (similar cDNA samples as were used for semiquantitative RT–PCR). All qPCRs were performed in duplo and run in an ABI/PRISM 7700 Sequence Detector System (Applied Biosystems) using the following conditions: 50°C for 2 min, 95°C for 10 min, and 40 cycles of 95°C for 15 s, 60°C for 1 min. To quantify the relative changes in gene expression of FABP7 and GDF15, we used the 2^-ΔΔ*C*T^ method (Winer *et al*, 1999; Livak and Schmittgen, 2001), in which 1F6 and Mel57 were selected as calibrator, respectively.

### Sequence analyses

The specificity of PCR products was validated by sequence analyses. Sequence reactions were performed with about 25 ng PCR product in addition of 10 *μ*M specific forward or reverse primer, using the ABI PRISM 3700 DNA Analyser (Perkin-Elmer, Applied Biosystems, Forster City, CA, USA).

### Immunohistochemistry

Cell suspensions of human melanoma cell lines 1F6 and Mel57 were processed into AgarCyto's as previously described (Kerstens *et al*, 2000). 4 *μ*m sections of paraffin-embedded AgarCyto's and melanocytic tumour lesions were mounted on Superfrost microscope slides. These sections were dewaxed in xylene and rehydrated in a series of graded alcohols. To block endogenous peroxidase activity, slides were incubated with 3% H_2_O_2_ for 20 min. In contrast to immunohistochemical detection of thrombomodulin (THBD), for GDF15 and FABP7 antigen retrieval was essential; rehydrated slides were placed in citrate buffer (pH 6.0) and heated in a microwave oven to 97°C at 850 W for 5 min. This temperature was maintained with an additional 10 min heating at 350 W. After cooling down to room temperature, the sections were briefly washed with PBS. Next to AgarCyto sections, also 1F6 and Mel57 cells that were cultured on slides were used for immunohistochemical staining. Therefore, cells were cultured to 70–80% confluency on Lab-Tek®II 8 Chamber slides (Nalge Nunc International Corp., Naperville), followed by fixation in acetone for 10 min. Prior to staining for THBD, GFD15 and FABP7, AgarCyto sections as well as cultured cells were subjected to a 20-min preincubation using 20% normal horse serum and 20% normal goat serum (Vector Laboratories, Burlingame, CA, USA), respectively. The sections/cells were stained in a three-step procedure utilising the following incubations: overnight incubation at 4°C with mouse monoclonal antibodies against THBD (DAKO-TM 1009) (DakoCytomation B.V., Heverlee, Belgium), rabbit polyclonal antibodies against GDF15 (US Biological, ImmunoSource, Zoersel-Halle, Belgium) or rabbit polyclonal antibodies against FABP7 (Cell Sciences Inc., Canton, MA, USA), diluted 1 : 50, 1 : 600 and 1 : 7500 in PBS, respectively. Thereafter, the sections/cells were incubated with a biotinylated horse-anti-mouse or goat-anti-rabbit antiserum for 30 min, followed by a 45-min incubation with peroxidase-labelled avidin-biotin complex (Vector Laboratories). Between all incubations, slides were washed three times in PBS. 3-amino-9-ethylcarbazole was used as substrate to visualise the bound antibodies. After counterstaining with Mayers haematoxylin, sections were mounted with Imsol.

### Western blotting

1F6 and Mel57 cells were solubilised using RIPA buffer (PBS, 1% Nonidet P40, 0.5% sodiumdeoxycholate, 0.1% SDS) containing Complete™, mini Protease Inhibitor Cocktail (Roche Diagnostics GmbH) and 2 mM PMSF. Equal amounts of proteins (40 *μ*g total cell lysate) were then separated on a 15% SDS polyacrylamide gel under reducing conditions. Subsequently, proteins were electrophoretically transferred onto a nitrocellulose membrane (Hybond ECL, Amersham Biosciences) in blot buffer (25 mM Tris pH 8.6, 0.2 M glycin and 20% methanol). Blots were incubated for 1 h in 0.5 × blocking buffer (LI-COR Biosciences, Bad Homburg, Germany)/PBS at room temperature, followed by an overnight incubation at 4°C with specific primary antibodies against GDF15 (see Immunohistochemistry section), diluted 1 : 1000 in blocking solution (0.5 × blocking buffer/PBS/0.05% Tween-20). After washing with PBS-T, blots were incubated for 1 h with goat-anti-rabbit-IRDye™ 800 infrared dye secondary antibodies (Rockland Immunochemicals Inc., Gilbertsville, PA, USA; dilution 1 : 2000 in blocking solution) under dark conditions, at room temperature. Then blots were scanned using the Odyssey Infrared Imager (LI-COR Biosciences).

## RESULTS

### Differential gene expression in human melanoma cell lines 1F6 and Mel57

The genes/ESTs that were spotted on our custom oligonucleotide array, were previously found to be differentially expressed in human melanoma cell lines 1F6 and Mel57 by using Affymetrix Hu6800 and Hu35K arrays (de Wit *et al*, 2002). To determine whether previous Affymetrix results could be reproduced using our custom made oligonucleotide arrays, we first performed a dye-swap experiment using Cy3 and Cy5 labelled cDNA of cell lines 1F6 and Mel57 as target probes for hybridisation. After scanning, we found that 20% (*n*=59) of the spots were not analysable. This was most probably due to insufficient quality of the oligo's, hybridisation efficiency and/or low copy expression of the genes in question. Of the 80% (*n*=239) that could be analysed, 98% (*n*=235) of the spots demonstrated differential gene expression that was identical to previous Affymetrix results. This showed that Affymetrix results were highly reproducible using our custom oligonucleotide array.

The indirect labelling method, which includes linear amplification of RNA, provided the opportunity to perform array analyses using a reduced amount of starting material. To determine validity of linear RNA amplification, target probes of cell lines 1F6 and Mel57 were prepared using this alternative labelling method and these probes were again hybridised to our custom arrays in a dye-swap experiment. Scanning of the arrays revealed that 58% (*n*=172) of the spots could be analysed, of which 99% (*n*=170) showed similar differential gene expression as was previously found by Affymetrix array analyses. Compared with the custom array experiment using the direct labelling method, we found that 91% (*n*=157) of the analysable genes showed a similar expression pattern and only one of these genes showed an aberrant differential expression than was found by Affymetrix arrays. Although our results demonstrated that a reduced number of spots could be analysed for differential gene expression using the indirect labelling method, the reproducibility of previous Affymetrix array results remained very high.

### Differential gene expression in human melanocytic lesions

As human melanoma cell lines 1F6 and Mel57 display different metastatic behaviour after subcutaneous inoculation into nude mice (Westphal *et al*, 1997), we reasoned that the genes that were found to be differentially expressed in these cell lines could well be involved in melanocytic tumour progression, especially in metastatic processes. To determine whether differential expression of these genes could also be detected in human melanocytic tumour lesions, we performed custom array analyses using target probes derived from NN and MM tissue samples. These NN and MM lesions contained a minimal tumour percentage of 50 and 60%, respectively. After Cy3 and Cy5 labelling of the probes using the indirect labelling method, individual NN and MM samples were hybridised in eight independent dye-swap experiments. Additionally, pooled samples of these NN (*n*=6) and MM lesions (*n*=6) were used to perform a dye-swap experiment. Compared to hybridisation experiments using cell line material, overall in the dye-swap experiments using melanocytic lesions, a lower percentage of the spots could be analysed, namely 15–40% (*n*=45–119). This can be explained by the considerably lower quality of total RNA of the lesions, influencing the labelling efficiency. In first instance, we determined differential expression only by visual analysis and by comparing the F-B values of the Cy dyes (GenePix Pro 4.0 software). In total, 42 genes/ESTs showed a highly reproducible differential gene expression, meaning similar results in more than five out of nine experiments. Although all of these genes might be candidate players in tumour progression, in this study we further focused on the 25 genes that showed comparable results in eight out of nine or even nine out of nine experiments (Table 2). For genes with similar differential expression in eight out of nine dye-swaps, we mostly found no detectable differential expression in the remaining experiment (no fluorescence signal or equal intensities for Cy3 and Cy5) and occasionally a swift in differential expression in NN and MM lesions (i.e. THBD, D4S234E, CTSL and GDF15).

To obtain an indication of the fold change of the differential gene expression, we also calculated the log_2_ of the median of ratios for the dye-swap experiments. Table 3 shows an example of these values for the dye-swap experiment using pooled NN and MM samples. For most of the genes, comparable fold changes could be determined within one dye-swap experiment, indicating the high reproducibility of the dye-swap arrays. However, log_2_ values were not always in accordance with data demonstrated in Table 2, as for instance is seen for NCK2 and ITGB5 genes in Table 3. Moreover, comparing all dye-swap experiments, we saw that although the differential expression in NN and MM was highly similar for the genes, the fold changes were quite variable. This suggests that the extent of differential gene expression is not necessarily the same when comparing different individual NN and MM lesions.

To determine whether among the 298 genes that were present on the array, differential gene expression also could be analysed in melanocytic tumour lesions of similar progression stages, we performed a dye-swap experiment using two different NN samples. Only eight genes showed differential expression, of which TYRP1 was one of them. The other seven genes did not belong to the group that showed differential gene expression comparing NN and MM in more than five dye-swap experiments. These eight genes most probably represent patient-specific and/or pigment-related differences in gene expression.

### Verification of differential gene expression

**Figure 2:**
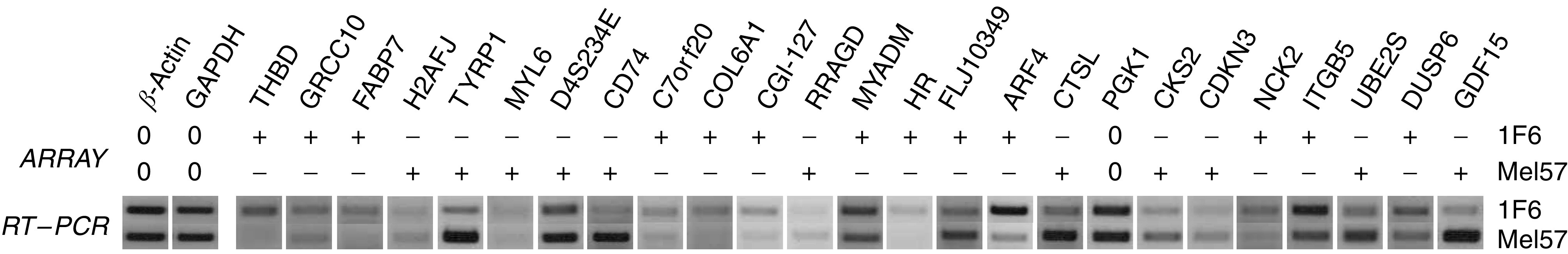
Verification of differential gene expression in human melanoma cell lines 1F6 and Mel57 by semiquantitative RT–PCR. Differential gene expression was determined by normalisation for *β*-actin and GAPDH. For nearly all of the 25 genes that were tested, differential expression that was found by custom array analyses could be validated by semiquantitative RT–PCR, except for MYL6 and FLJ10349.

**Figure 3:**
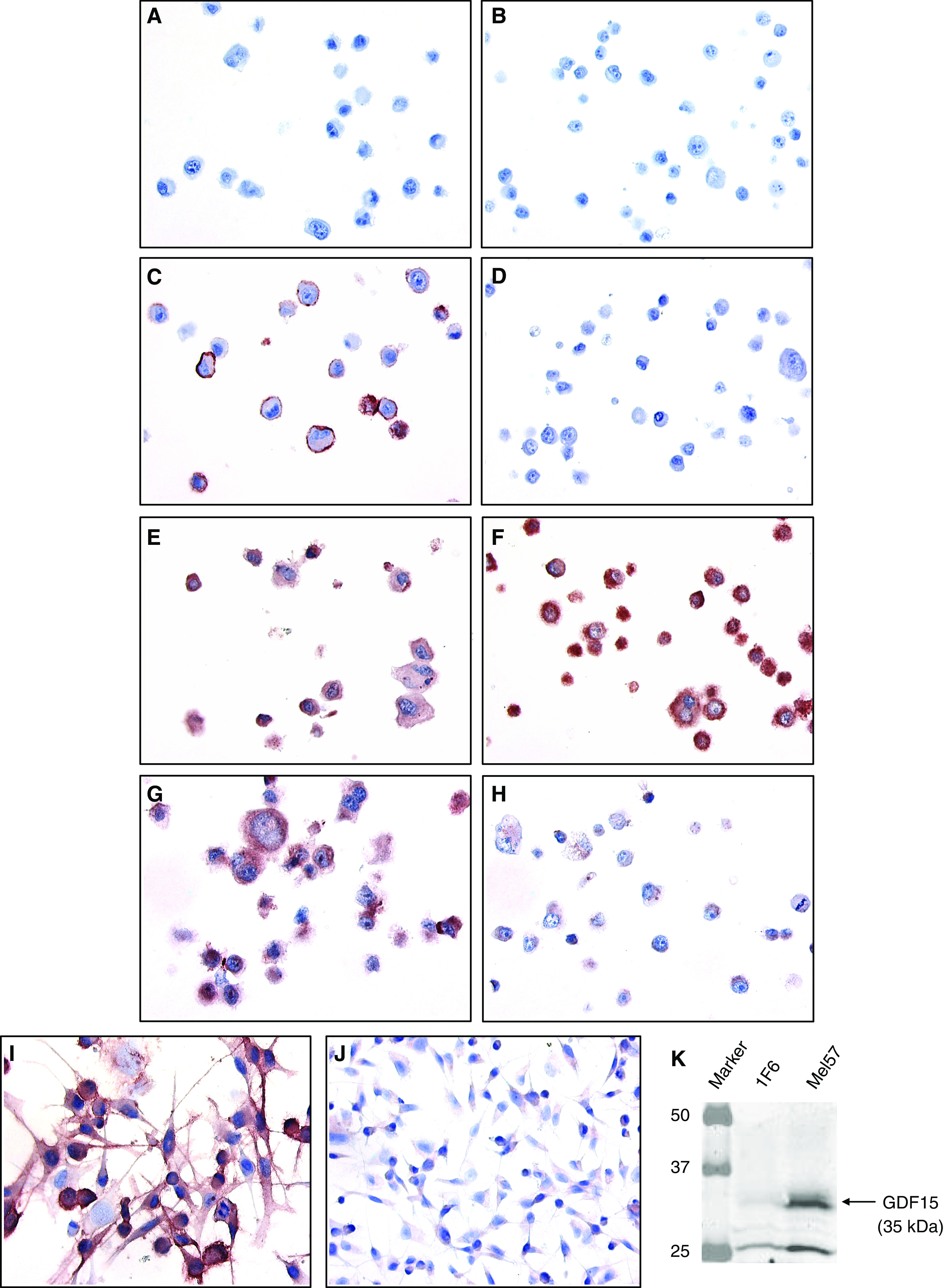
Analysis of protein expression of THBD, GDF15 and FABP7 in human melanoma cell lines 1F6 and Mel57. Magnification (× 400) of AgarCyto's (**A–H**) and cultured 1F6 and Mel57 cells (**I, J**). (**A, B**) Negative control of 1F6 and Mel57, respectively, leaving out specific antisera. (**C, D, I, J**) IHC using THBD specific antibodies; in AgarCyto's as well as cultured cells most 1F6 cells showed a (membranous) THBD specific staining (**C, I**), whereas Mel57 cells were completely negative (**D, J**). (**E, F**) IHC using GDF15 specific antibodies; a more intense cytoplasmic GDF15 staining could be detected in cell line Mel57 (**F**), compared to 1F6 cells (**E**). (**G, H**) IHC using FABP7 specific antibodies; 1F6 cells showed a higher immunoreactivity for FABP7 (cytoplasmic) (**G**) than Mel57 cells (**H**). (**K**) Western blot analysis of GDF15; a distinct preferential protein expression could be detected in Mel57 compared to 1F6. The 35 kDa band represents the pro-form of GDF15 (pro-peptide+mature protein), whereas the 25 kDa band shows the cleaved GDF15 pro-peptide.

To validate the differential gene expression we found by custom array analysis, we performed semiquantitative RT–PCR for the 25 genes indicated in Table 2. We first looked at their differential expression found in melanoma cell lines 1F6 and Mel57 (Figure 2). Using GAPDH and *β*-actin for normalisation, we determined that differential expression could be corroborated for all genes, except for MYL6 and FLJ10349. PGK1, which was initially selected as housekeeping gene, showed indeed no differential expression in the melanoma cell lines. By performing immunohistochemistry (IHC) on AgarCyto's of 1F6 and Mel57, we wanted to determine whether differential expression could also be detected on the protein level. Unfortunately, antibodies were only limitedly available. Nevertheless, IHC using THBD, GDF15 and FABP7 specific antibodies showed that a similar differential protein expression could be detected as was seen for their mRNA expression by array analyses and semiquantitative RT–PCR (Figure 3). Figure 3C and D shows that THBD was mainly localised to the cell membrane in 1F6 cells, whereas Mel57 cells were completely negative. For GDF15 the difference in protein expression was less distinctive; however, an upregulated cytoplasmic expression could be detected in cell line Mel57 compared to 1F6 (Figure 3E and F). FABP7 showed a significant higher cytoplasmic protein expression in 1F6 than could be detected in Mel57 (Figure 3G and H). Next to AgarCyto's, also slides containing cultured 1F6 and Mel57 cells were stained for THBD, GDF15 and FABP7. These cultured cells showed comparable staining results as were found with IHC on AgarCyto's. Even though the observed differences in protein expression for FABP7 and especially for GDF15 were a little less profound in the cultured cells (data not shown), THBD again showed a marked expression in 1F6, whereas Mel57 cells remained negative (Figure 3I and J). The differential protein expression of GDF15 could be better visualised by Western blot analysis as is demonstrated in Figure 3K. In this figure, the 35 kDa band represents the pro-form of GDF15 (propeptide+mature protein), whereas the 25 kDa band shows the cleaved GDF15 propeptide. The mature form of GDF15 (10 kDa) could hardly be detected by Western blotting using cell lysates as this protein is secreted by the cells.

**Figure 4:**
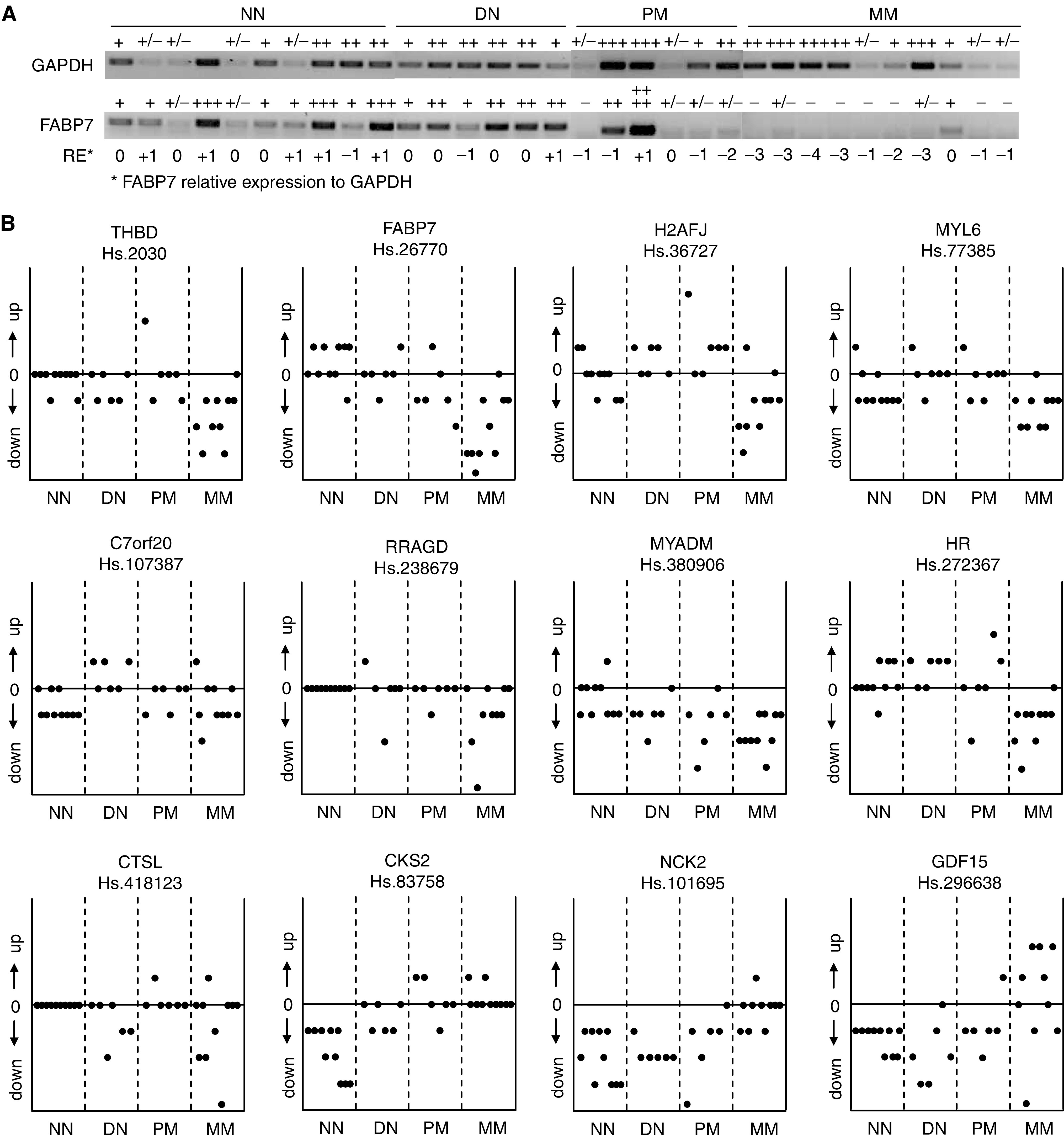
Verification of differential gene expression in human melanoctyic tumour progression lesions by semiquantitative RT–PCR. (**A**) For 12 differentially expressed genes, relative expression to GAPDH was determined by semiquantitative RT–PCR as shown for FABP7. (**B**) For MYL6 and C7orf20 differential expression could hardly be detected. THBD, FABP7, H2AFJ, RRAGD, MYADM and HR showed a downregulated expression during melanoma progression, with a preferable decrease in MM lesions. The latter was especially true for H2AFJ. Expression of CKS2, NCK2 and GDF15 showed an upregulation associated with increased malignancy of melanocytic tumour lesions. For THBD, FABP7, H2AFJ, RRAGD, MYADM, HR, CKS2, NCK2 and GDF15 differential expression found by semiquantitative RT–PCR was in accordance with our custom array data, whereas CTSL showed an inverse differential expression.

Also for human melanocytic tumour lesions, semiquantitative RT–PCR was used to verify differential gene expression. As only a limited amount of cDNA was available of most lesions, a pilot experiment was performed in which differential expression of the 25 genes listed in Table 2 was determined in two samples of NN, atypical nevi (AN), primary melanoma (PM) and MM. This experiment showed that 12 of the 25 genes displayed distinct differential expression during melanoma progression (data not shown). These 12 genes were selected for further expression profiling in a larger series of melanocytic tumour lesions. For all 12 genes, the relative expression to GAPDH was determined as is demonstrated for FABP7 in Figure 4A. Figure 4B shows the graphical representation of the relative expression of these 12 genes during melanoma progression. For MYL6 and C7orf20, no distinct differential expression could be detected in this larger series and CTSL showed an inversed differential expression by semiquantitative RT–PCR than was found by custom array analyses. In accordance with our array data, downregulation of THBD, FABP7, H2AFJ, RRAGD, MYADM and HR and upregulation of CKS2, NCK2 and GDF15 was seen in MM compared to NN lesions. The upregulation of CKS2, NCK2 and GDF15 seemed to follow a somehow gradual course during melanocytic tumour progression, whereas downregulated expression of THBD, FABP7, RRAGD, MYADM, HR and especially H2AFJ was more predominantly restricted to MM lesions. To exclude that differential expression was significantly influenced by the presence of keratinocytes in the NN, AN and PM lesions, we performed semiquantitative RT–PCR on two normal skin (NS) and laser dissected melanocytic cells derived from two NN lesions. Only for C7orf20 and MYADM, we found that the presence of keratinocytes most probably caused the differential expression seen by array analyses and RT–PCR. Overall, it has to be noted that for some differentially expressed genes no PCR product could be detected after 35 cycles, meaning that expression of these genes might even be stronger downregulated in the melanocytic tumour lesions than is demonstrated in Figure 4B.

**Figure 5:**
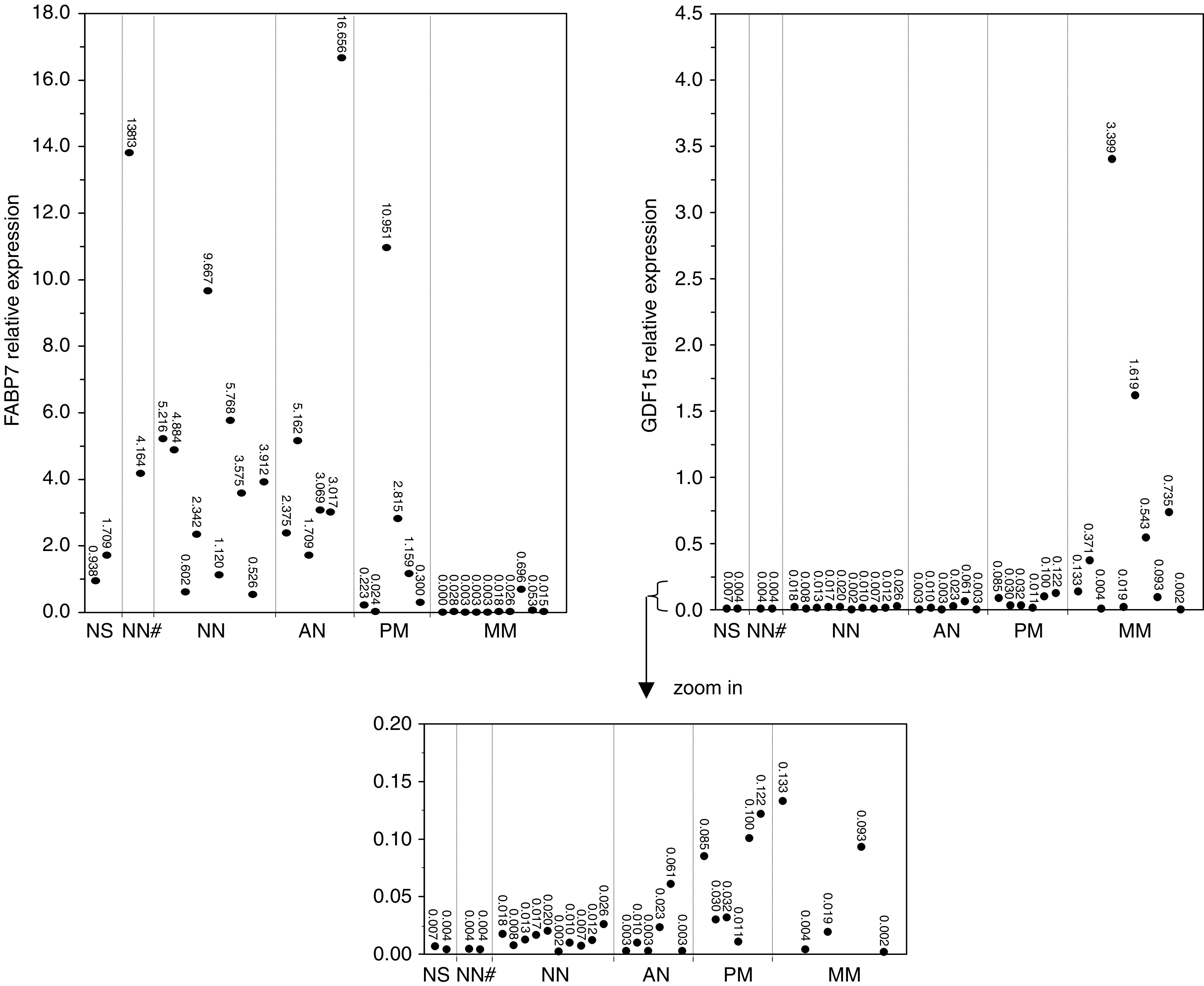
Verification of differential gene expression of FABP7 and GDF15 in human melanoctyic tumour progression lesions by real-time qPCR. For FABP7 as well as GDF15, GAPDH was used for normalisation. FABP7 expression in melanocytic tumour lesions is demonstrated relative to expression in cell line 1F6. A downregulated FABP7 expression was detected during melanocytic tumour progression, which was most prominent in MM lesions. For GDF15, expression in melanocytic tumour lesions is demonstrated relative to expression in cell line Mel57. GDF15 showed an upregulated expression in malignant stages of melanocytic tumour progression. Inclusion of normal skin (NS) tissue and laser dissected melanocytes derived from NN lesions (NN#) in the real-time qPCR analyses revealed that epidermal cells do not significantly influence the differential gene expression of FABP7 and GDF15 that is seen in NN and MM lesions.

To strengthen our semiquantitative RT–PCR results and more precisely quantify the differences found in expression, we performed real-time qRT–PCR for two randomly selected genes, namely FABP7 and GDF15. We first determined fold change of differential expression between cell line 1F6 and Mel57. A 24-fold increase of FABP7 expression was found in 1F6 compared to Mel57; for GDF15 a 10-fold decrease was detected. Figure 5 demonstrates the expression of FABP7 and GDF15 in melanocytic tumour lesions relative to 1F6 and Mel57, respectively. qPCR results were highly similar to our data derived from custom array analyses and semiquantitative RT–PCRs. For FABP7 a decreased expression could again be determined when comparing MM lesions to NN lesions. The opposite was true for GDF15. During tumour progression, we found that down- or upregulated expression of FABP7 and GDF15, respectively, could already be detected in PM lesions, but was most prominent in MM. Moreover, NS and laser dissected NN samples (NN#) that were included in this qPCR ensured that keratinocytes did not influence the differential gene expression of FABP7 and GDF15 in NN and MM lesions. As for some tumour samples again no PCR product could be detected after 40 cycles, downregulated expression shown in Figure 5 might probably be underestimated.

To provide an indication of the overall fold change of FABP7 and GDF15 differential expression in NN and MM lesions, we calculated the average relative expression of NN and MM samples obtained by qPCR and compared these values. We found a 164-fold downregulation of FABP7 expression in MM compared to NN, whereas GDF15 showed a 53-fold upregulated expression. These fold changes are much higher than those found by custom array analyses (Table 3).

## DISCUSSION

In this study, custom made oligonucleotide arrays were designed, based on previous Affymetrix high-density oligonucleotide array results, in which differential expression was analysed in human melanoma cell lines 1F6 and Mel57 (de Wit *et al*, 2002). The highly comparable results obtained by both array techniques, concerning differential gene expression in 1F6 and Mel57, indicate that our custom arrays are a reliable tool for analysis of differential gene expression.

Previous studies already showed that linear RNA amplification provides the opportunity to perform array analysis with minute amounts of starting material (Puskas *et al*, 2002; Gomes *et al*, 2003; Klur *et al*, 2004; Schneider *et al*, 2004). We experienced that the indirect labelling method, which is based on linear RNA amplification, is indeed a valuable and reliable technique, although we determined a reduction in number of analysable spots. This might be explained by lack of optimal amplification efficiency, as the quality of total RNA can negatively influence the procedure. However, we found that signal intensities of the spots that could be analysed were generally hardly diminished. Besides linear RNA amplification by *in vitro* transcription (IVT), alternative methods have recently been described to reduce the amount of staring material for array analysis. For instance, amplification of full-length double-stranded cDNA by PCR has been shown to be useful in various studies (Saghizadeh *et al*, 2003; Becker *et al*, 2004; Rihl *et al*, 2004), and also application of single-stranded linear amplification protocol (SLAP) has been reported, which combines linear amplification and PCR (Stirewalt *et al*, 2004). However, it has to be noted that for identification of differential gene expression by array analysis, it is highly preferable that each sample is equally treated prior to hybridisation, as every amplification method has the possibility to introduce transcript-dependent biases, which even increase as the starting amount of RNA decreases (Stirewalt *et al*, 2004).

Using fresh NN and MM lesions for custom array analyses, we determined whether the differential gene expression that was previously found in melanoma cell lines 1F6 and Mel57 could also be detected in the *in vivo* situation. Differential gene expression was analysed in two ways: by comparing F-B values of the Cy dyes and by calculating the log_2_ of the median of ratios for each dye swap experiment. For the latter method, which provided an indication of the fold change of the differential gene expression, the ‘median of ratios’ was chosen over the commonly used ‘ratio of medians’ as Brody *et al* (2002) determined that the ‘median of ratios’ provided a more consistent measurement. However, as the size of the spots still influences the accuracy of the measurement of the ‘median of ratios’ values (Brody *et al*, 2002), in our study we attached higher importance to the F-B software analysis. Moreover, comparison of F-B values of the Cy dyes showed a better correlation with visual analysis of the arrays. In this way, 25 genes were identified showing a highly reproducible constant differential expression pattern in NN *vs* MM lesions. The dye-swap experiment using two NN samples for hybridisation showed that the differential expression of most of these genes was not patient specific.

For nine of the 25 genes, namely THBD, FABP7, H2AFJ, RRAGD, MYADM, HR, CKS2, NCK2 and GDF15, our semiquantitative RT–PCR results were in accordance with the differential expression that was found by custom array analyses. However, the possibility remains that for some of the 13 genes that were now only tested in a pilot experiment using semiquantitative RT–PCR, verification of differential expression can still be achieved using an expanded series of melanocytic tumour lesions. For instance, DUSP6 was very recently also found to be differentially expressed in normal melanocytes compared to melanoma cells by Hoek *et al* (2004). This indicates it might be an interesting gene for further studies. Additionally, also genes that showed a differential expression in five out of nine, six out of nine and seven out of nine custom array experiments might still be potential players in melanocytic tumorigenesis.

Real-time qPCR, performed for FABP7 and GDF15, strengthened the data of our semiquantitative RT–PCR analyses, as both techniques provided comparable results. Moreover, it confirmed differential gene expression found by custom array analysis, although fold changes that were found by performing qPCR were much higher than those obtained using arrays (log_2_ of the median of ratios, Table 3). The tendency to underestimate fold change ratios by array analysis is also reported in other studies (Rajeevan *et al*, 2001; Yuen *et al*, 2002).

Our differential gene expression data showed minimal overlap with previously described microarray experiments in which also gene expression patterns were studied in cutaneous melanoma progression (Brem *et al*, 2001; Baldi *et al*, 2003b; Carr *et al*, 2003; Dooley *et al*, 2003; Rumpler *et al*, 2003; Becker *et al*, 2004; Nambiar *et al*, 2004). This could be explained by the fact that design of our custom made oligonucleotide arrays was based on previously found differential gene expression in melanoma cell lines. This way, we already made a considerable selection of genes to be examined for differential gene expression, probably missing some genes that might also be involved in melanocytic tumour progression. Moreover, high variability can be introduced between related array studies, as arrays designed with different types of oligonucleotides/cDNAs (e.g. 50-/60-/70-mer oligo's, full-length cDNA) can be used and distinct methods can be selected for labelling of target probes. Therefore, numerous comparable microarray studies are described with different outcomes (Nambiar *et al*, 2004). This emphasises the necessity of verification of the array data using more conventional techniques, like (semi-)quantitative RT–PCR, to prove that differential gene expression is really present.

Until now, minimal information is available in literature of most of the genes that we found differentially expressed by our custom array analyses as well as by RT–PCR. However, GDF15 and THBD were previously described to be involved in tumorigenesis. GDF15, which is also called macrophage inhibitory cytokine-1 (MIC-1), NSAID-activated protein (NAG-1) or prostate differentiation factor (PLAB), is a divergent member of the tumour growth factor *β* (TGF-*β*) superfamily (Bootcov *et al*, 1997; Hromas *et al*, 1997; Baek *et al*, 2001). The major function of GDF15 is still uncertain, but there are indications that it plays a role in growth inhibition and induction of apoptosis in several tumour cell lines (Li *et al*, 2000; Albertoni *et al*, 2002; Yang *et al*, 2003). Controversially however, several studies have reported an upregulated (secreted) expression in (advanced and more aggressive) tumours compared to noncancerous tissues or less aggressive tumours (Welsh *et al*, 2001; Brown *et al*, 2003; Karan *et al*, 2003; Nakamura *et al*, 2003). This is in accordance with our data, as we also found an upregulated expression during melanoma progression. THBD is a thrombin receptor, which is mostly found on the surface of vascular endothelial cells and epidermal keratinocytes. However, its presence is also reported on tumour cells of several types of cancer, such as, hepatocellular carcinoma, ovarian cancer, breast cancer and squamous cell carcinoma (Suehiro *et al*, 1995; Kim *et al*, 1997; Tabata *et al*, 1997; Wilhelm *et al*, 1998). In these tumours, the expression level of THBD is inversely correlated with malignancy of cancer. As for melanoma, it was previously reported that THBD isolated from human urine could suppress experimental lung metastasis of murine melanoma cells (B16F10 cells) in mice and it inhibited invasion of these cells *in vitro* (Hosaka *et al*, 2000). Also for human melanoma cell lines, a negative correlation was described between THBD expression and cell proliferation *in vitro* and *in vivo* (Zhang *et al*, 1998). This is in accordance with our findings, as we determined a downregulated THBD expression in MM lesions by array analysis and RT–PCR. Moreover, our IHC data showed membranous expression in human melanoma cell line 1F6, whereas the more malignant cell line Mel57 remained negative. Although, previous studies report THBD expression in keratinocytes, our RT–PCR data of NS and laser dissected melanocytic NN cells indicated that epidermal cells were not responsible for the differential expression that we determined in NN and MM lesions. Obviously, for both GDF15 and THBD, additional studies are necessary to elucidate their role in cancer biology and determine its potential clinical significance.

In summary, this study provides a reliable, solid indication that CKS2, NCK2, GDF15 THBD, FABP7, RRAGD, MYADM, HR and H2AFJ are candidate players in melanocytic tumour progression, as similar differential expression patterns are demonstrated for these genes by custom array analysis, semiquantitative RT–PCR and even real-time qPCR. The genes that showed a preferable expression in benign melanocytic tumour stages and loss of expression during tumour progression might be functional markers to facilitate early diagnosis of melanocytic lesions before onset of the metastatic phase. Genes with an elevated expression in advanced, more malignant melanocytic lesions are probably more suitable for development of novel therapeutics for melanoma patients. Moreover, regarding prognostic settings, the downregulated genes can be potential indicators for a relatively good prognosis, whereas the upregulated genes might be of opposite prognostic value. Nevertheless, to ensure that the genes that we found to be differentially expressed in this study are indeed useful for diagnostic, prognostic and/or therapeutical applications, further investigation is necessary.

## Figures and Tables

**Table 1:** Primers used for semiquantitative RT–PCR analyses

**Gene symbol**	**Primers**	**MgCl_2_ (mM)**
THBD	F: 5′-GTGGACGGCGAGTGTGTG-3′	1.5
	R: 5′-CAGAGGTAGCTAGTTTGGTTCAGG-3′	
GRCC10	F: 5′-TCGTGCTGCCCGTGG-3′	1.5
	R: 5′-AGGCTGGCGATCTCAGGAT-3′	
FABP7	F: 5′-AACTTGTTCACATACAGAAATGGGAT-3′	2.0
	R: 5′-AGAACATTTTTATGCCTTCTCA-3′	
H2AFJ	F: 5′-CGTGCTGCTGCCCAAGA-3′	1.5
	R: 5′-TTGCGGGACGACCATGA-3′	
TYRP1	F: 5′-CTTGGAAGATTATGATACCCTGGG-3′	1.5
	R: 5′-GAGCGACATCCTGTGGTTCA-3′	
MYL6	F: 5′-GAAGCGTTTGTGAGGCATATCC-3′	2.0
	R: 5′-TAGATACAAAATTCACACAGGGAAAGG-3′	
D4S234E	F: 5′-TTCCTCACCTGCGTCGTCTT-3′	1.5
	R: 5′-CGTAGTAGCTCTCCAAGCGTTCTG-3′	
CD74	F: 5′-GAAGATCAGAAGCCAGTCATGG-3′	1.5
	R: 5′-AGAGCTACCAGGATGGAAAAGC-3′	
C7orf20	F: 5′-GCCGCTGCTTAACTTCATCTG-3′	1.5
	R: 5′-GTCCTATGCGGTCGAGGTACTC-3′	
COL6A1	F: 5′-GCGACGCACTCAAAAGCA-3′	1.5
	R: 5′-GGTACTTATTCTCCTTCAGGTGGG-3′	
CGI-127	F: 5′-GACCAACCGCTCAGAACTCATC-3′	1.5
	R: 5′-AGCATGACCCGASTCTTGAACTT-3′	
RRAGD	F: 5′-ATTGACTTTTTTGACCCTACATTTGAC-3′	1.5
	R: 5′-GCCAGGGCTTCCATGTAATC-3′	
MYADM	F: 5′-GTGGCTCAATCCGTCTCCA-3′	2.0
	R: 5′-GATGGTGGTGCGGGTTACC-3′	
HR	F: 5′-CAAGGATGTGGACTCGGGA-3′	1.5
	R: 5′-CAGTTTTGCAGGGAGAGCCA-3′	
FLJ10349	F: 5′-GCCACCATGCTGCACGT-3′	1.5
	R: 5′-GTGGTCAAGCAGGTAGGAGAGG-3′	
ARF4	F: 5′-CCCTCTTCTCCCGACTATTTGG-3′	1.5
	R: 5′-ATTGGTGGTGACTATCTCCCCTAAC-3′	
CTSL	F: 5′-CTGTAGCAGTGAAGACATGGATCA-3′	1.5
	R: 5′-CGTAGCCACCCATGCCC-3′	
PGK1	F: 5′- TAAAGGGAAGCGGGTCGTT-3′	1.5
	R: 5′-GTGGCTCATAAGGACTACCGACTT-3′	
CKS2	F: 5′-CGCTCTCGTTTCATTTTCTGC-3′	1.5
	R: 5′-TGGAAAGTTCTCTGGGTAACATAACA-3′	
CDKN3	F: 5′-TACAACCTGCCTTAAAAATTACCGA-3′	1.5
	R: 5′-GACAGGTATAGTAGGAGACAAGCAGCT-3′	
NCK2	F: 5′-GGCTATGTACCGTCCAACTACG-3′	1.5
	R: 5′-GCTGGTCTTCCTGCGC-3′	
ITGB5	F: 5′-GAGGAAGTGTGAGGGTCTGAAGA-3′	1.5
	R: 5′-TGACCCCCACCTCCAGGCT-3′	
UBE2S	F: 5′-CCAGGTCACCATCGAGGG-3′	1.5
	R: 5′-CTTCCCCAGCAGGAGTTTCA-3′	
DUSP6	F: 5′-CCTTCCTTCCCAGTGGAGATC-3′	2.0
	R: 5′-CTCAAAGAGATTCGGCAAATTGG-3′	
GDF15	F: 5′-CGAAGACTCCAGATTCCGAGAG-3′	1.5
	R: 5′-CCAGCCGCACTTCTGGC-3′	
GAPDH	F: 5′-CGACAGTCAGCCGCATCTT-3′	1.5
	R: 5′-GCCCAATACGACCAAATCCG-3′	
*β*-Actin	F: 5′-CGTGCTGCTGACCGAGG-3′	1.5
	R: 5′-GCAACGTACATGGCTGGGG-3′	

**Table 2:** Differential gene expression between NN and MM samples determined by dye-swap array experiments

**Table 3:** Fold changes of differentially expressed genes in a dye-swap experiment using pooled NN and MM samples
